# Supplementary material for: Conditional chemoconnectomics (cCCTomics) as a strategy for efficient and conditional targeting of chemical transmission
Source: eLife. 2024 Apr 30;12:RP91927. doi: 10.7554/eLife.91927 (PMC11060718; doi:10.7554/eLife.91927)
Supplement: Figure 3—source data 3. [file elife-91927-fig3-data3.zip › Figure 3 source data3 part 1.zip!/Figure 3-Source data 3-part1/1071-1-CRISPResso_Report_osfbjvga.zip!/CRISPResso_on_1071_3/9.Alleles_frequency_table_around_sgRNA_gaccgtctcaccgtcaagat.pdf]

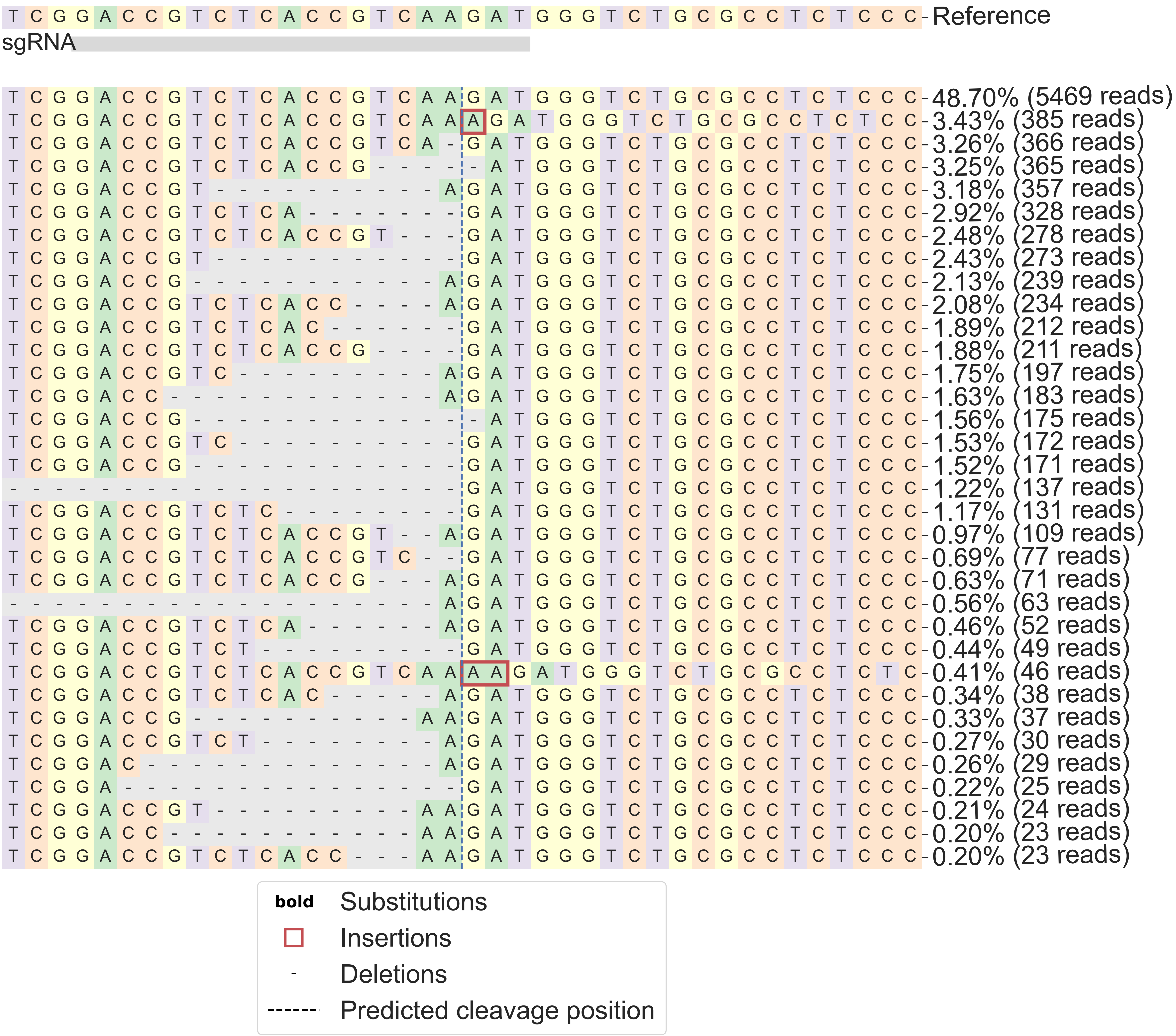

TCGGACCGTCTCACCGTCAAGATGGGTCTGCGCCTCTCCC
Reference
sgRNA
48.70% (5469 reads)
3.43% (385 reads)
3.26% (366 reads)
3.25% (365 reads)
3.18% (357 reads)
2.92% (328 reads)
2.48% (278 reads)
2.43% (273 reads)
2.13% (239 reads)
2.08% (234 reads)
1.89% (212 reads)
1.88% (211 reads)
1.75% (197 reads)
1.63% (183 reads)
1.56% (175 reads)
1.53% (172 reads)
1.52% (171 reads)
1.22% (137 reads)
1.17% (131 reads)
0.97% (109 reads)
0.69% (77 reads)
0.63% (71 reads)
0.56% (63 reads)
0.46% (52 reads)
0.44% (49 reads)
0.41% (46 reads)
0.34% (38 reads)
0.33% (37 reads)
0.27% (30 reads)
0.26% (29 reads)
0.22% (25 reads)
0.21% (24 reads)
0.20% (23 reads)
0.20% (23 reads)
bold Substitutions
Insertions
Deletions
Predicted cleavage position
